# *Bartonella*-Associated Transverse Myelitis

**DOI:** 10.3201/eid2304.161733

**Published:** 2017-04

**Authors:** Parham Sendi, Cedric Hirzel, Andreas Bloch, Urs Fischer, Natalie Jeannet, Livia Berlinger, Heinz Krestel

**Affiliations:** Bern University Hospital, Bern, Switzerland; University of Bern, Bern; Bioanalytica AG, Luzern, Switzerland

**Keywords:** myelitis, transverse myelitis, *Bartonella henselae*, cat-scratch disease, bacteria, zoonoses

## Abstract

Each year in the United States, 500 patients are hospitalized for cat-scratch disease, caused by *Bartonella henselae* infection. We report a case of rare but serious neurologic *B. henselae* infection. When typical features of cat-scratch disease occur with neurologic findings, *Bartonella* infection should be suspected and diagnostic testing should be performed.

In a recent epidemiologic study, Nelson et al. (*1*) estimated that each year in the United States, 500 patients are hospitalized for cat-scratch disease (CSD), caused by *Bartonella henselae* infection. Typical disease presentation includes enlarged lymph nodes proximal to the site of organism inoculation. *B. henselae* can disseminate and infect various organs, including the central nervous system (CNS). For patients with neurologic involvement, laboratory diagnosis can be challenging.

In 2015, a previously healthy 46-year-old woman was referred to Bern University Hospital, Bern, Switzerland, for suspected acute ischemic stroke; she was experiencing dysarthria, aphasia, dysphagia, paresthesia, and weakness in both legs. She reported no travel history or recent vaccination but reported having had contact with her neighbor’s cat.

Physical examination revealed blood pressure 130/80 mm Hg and heart rate 100 beats/min. Also noted were flaccid paralysis of the lower extremities (manual muscle testing score 4, dorsal and plantar flexion of the foot; manual muscle testing score 2, flexion and extension of the thighs), dysarthria, peripheral facial paralysis, and gaze-evoked nystagmus. Serum leukocyte count was 7.2 × 10^9^ cells/L (without a left shift of neutrophils), and serum C-reactive protein level was 8 mg/L (reference <5 mg/L). Magnetic resonance images of the brain showed no abnormalities, but those of the spinal cord showed longitudinal lesions consistent with transverse myelitis (Figure). Analyses of cerebrospinal fluid (CSF) revealed a cell count of 167/μL (98% mononuclear cells) and elevated levels of protein (1.1 g/L) and lactate (2.4 mmol/L). The CSF/serum ratio of albumin indicated a blood–CSF barrier dysfunction. Thus, a diagnosis of meningoencephalitis and acute transverse myelitis was made. 

**Figure:**
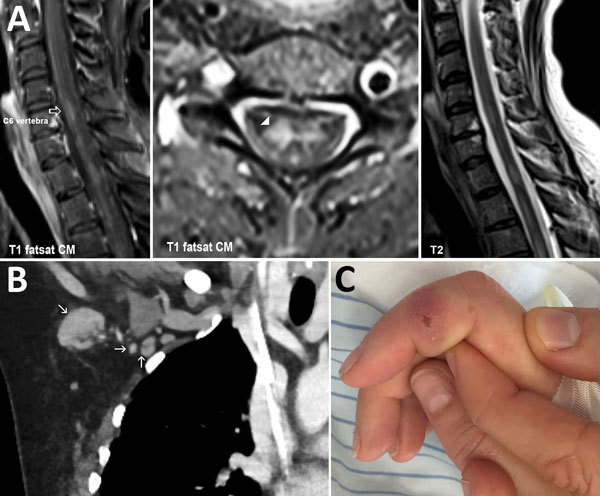
Images of woman with transverse myelitis and *Bartonella henselea* infection. A) Magnetic resonance image of the spine showing transverse myelitis (arrowhead). Fat-saturated (fs) T1-weighted image with contrast medium (cm), sagittal plane (left panel) and axial plane (middle panel). T2-weighted image, sagittal plane (right panel). B) Coronal view of computed tomography image of the chest, showing right axillar lymphadenopathy (arrows). C) Right index finger, showing a persistent ulcer from a cat scratch.

Initial treatment consisted of corticosteroids and empirically prescribed antiinfective therapy with acyclovir, amoxicillin, and ceftriaxone. After 5 days of incubation, CSF samples showed no microorganism growth; however, specific culture techniques for *Bartonella* spp. were not used because lumbar puncture had been performed before CSD was suspected. PCR results were negative for herpes simplex virus 1/2, varicella zoster virus, cytomegalovirus, Epstein-Barr virus, and enterovirus. Serologic test results were negative for *Borrelia burgdorferi, Treponema pallidum*, *Mycoplasma pneumoniae*, tickborne encephalitis virus, *Toxoplasma gondii*, and HIV, as were results for *Cryptococcus neoformans* serum antigen testing. Results of a multiplex PCR for respiratory viruses and *M. pneumoniae* in a nasopharyngeal swab sample were negative. Therefore, empirical treatment with antiviral and antibiotic agents was stopped. Because no infectious etiology was found, the differential diagnosis included vasculitis and neoplastic and paraneoplastic disorders. The clinical findings and high CSF cell count argued against multiple sclerosis. Test results were negative for autoantibodies (antinuclear antibodies; c and p antineutrophil cytoplasmic antibodies; double-stranded DNA antibodies; and antiphospholipid, onconeural, and neuromyelitis optica [antiaquaporin 4] autoantibodies). 

Computed tomography of the chest and abdomen showed no evidence of neoplasia but did show enlarged right-sided axillary lymph nodes (Figure). This result, together with a visible scratch on the patient’s right index finger (Figure) and a history of contact with the neighbor’s cat, was highly suggestive of CSD. The PCR result for *B. henselae* (Technical Appendix) in a biopsy sample from the right index finger was positive, as was the result of an indirect immunofluorescence assay for *B. henselae* IgG (titer 1:512). After being asked, the patient reported having been scratched by the cat 6 weeks earlier and having felt enlarged axillary lymph nodes 4 weeks before admission. The PCR result for *B. henselae* in CSF was negative. Intrathecal antibody production was not elevated, and the specific CSF/serum ratio for IgG against *Bartonella* was negative, although the laboratory method for the latter is not standardized. CSD-associated transverse myelitis was postulated. Doxycycline was given for 3 weeks and continued with tapering doses of corticosteroids. The patient improved, but at follow-up examination 6 months after discharge, she reported residual neurologic symptoms, including fatigue, chronic headache, radicular neuropathic pain, and slight gait unsteadiness. *B. henselae* IgG titer was 1:64.

For this patient, the classic features of CSD were present, and laboratory diagnosis of CSD was made by 2 methods. Nonetheless, we detected neither *Bartonella* antigens in CSF via PCR nor significant intrathecal production of antibodies against *Bartonella*. Thus, we cannot rule out coincidental CSD and idiopathic inflammatory myelitis. However, in the latter disease, the CSF cell count is typically markedly lower and the myelitis less extensive than was observed for this patient. Moreover, the consistent time course of the disease, lack of an alternative diagnosis, and similarity to other clinical courses suggest CSD-associated transverse myelitis (*2*). In previously published cases of patients with CNS manifestations and CSD, laboratory diagnosis was not made from CSF samples (*2**–**6*). Therefore, it is uncertain whether the pathogenesis of myelitis is the result of direct invasion of the spinal cord by *B. henselae* or an immune-mediated postinfectious process (*2*).

Our report and others (*2**–**6*) demonstrate that diagnosis of *B. henselae* CNS disease is currently based on neurologic symptoms and findings after a cat scratch, not on laboratory diagnosis of CSF or CNS biopsy samples. Nonetheless, for suspected cases of CSD, laboratory studies from serum or skin lesion samples should be used for confirmation.

Technical Appendix Primers and probes used to detect *Bartonella henselae* in study of *Bartonella*-associated transverse myelitis.
