# Supplementary material for: Bartonella-Associated Transverse Myelitis
Source: Emerg Infect Dis. 2017 Apr;23(4):712–3. doi: 10.3201/eid2304.161733 (PMC5367412; doi:10.3201/eid2304.161733)
Supplement: Technical Appendix — Primers and probes used to detect Bartonella henselae in study of Bartonella-associated transverse myelitis. [file 16-1733-Techapp-s1.pdf]

Article DOI: http://dx.doi.org/10.3201/eid2304.161733

Bartonella-Associated Transverse Myelitis

Technical Appendix

Primers and probes used in the case to detect *Bartonella henselae*, modified from (*1,2*):

Primers

CAT-1: GAT TCA ATT GGT TTG AA(G/A) GAG GCT

CAT-2: CAA TAC GCT TTG CTA GAT CAC G

Gene Probes

B. henselae (Bh1-FAM): FAM - TGC GTT AAT TAC CGA TCC ATT AAA GGR GCC - BHQ1

B. quintana: YAK - CGC TTT GAT TAC TGA TCC GTT AAA GGG T - BBQ

A two-probe Taqman real-time PCR was performed to differentiate between *B. henselae* and *B. quintana* targeting the *htrA* gene of *Bartonella* sp.

References

1. Anderson B, Sims K, Regnery R, Robinson L, Schmidt MJ, Goral S, et al. Detection of Rochalimaea henselae DNA in specimens from cat scratch disease patients by PCR. J Clin Microbiol. 1994;32:942–8. PubMed

2. Goldenberger D, Zbinden R, Perschil I, Altwegg M. [Detection of Bartonella (Rochalimaea) henselae/B. quintana by polymerase chain reaction (PCR)]. Schweiz Med Wochenschr. 1996;126:207–13. PubMed
